# Association of HIV infection and alcohol use on resting-state functional connectivity and mental health in youth living with perinatal HIV

**DOI:** 10.21203/rs.3.rs-9266867/v1

**Published:** 2026-06-05

**Authors:** Aliaa Ibnidris, Freda Scheffler, Jonathan Ipser, Heather J Zar, Dan J Stein, Jackie Hoare

**Affiliations:** University of Cape Town

**Keywords:** Youth, perinatal HIV, alcohol use, rs-fMRI, functional connectivity, depression, anxiety

## Abstract

**Background:**

Youth living with perinatally acquired HIV (YPHIV) are at increased risk for developing mental health disorders, particularly depression and anxiety, which may be compounded by alcohol use. Research in adults living with HIV indicates that both HIV infection and alcohol use have been linked to altered resting-state functional connectivity (RSFC) in limbic and striatal circuits, and that this, in turn, is associated with severity of anxiety and depressive symptoms. However, the combined effects of perinatal HIV infection, alcohol use and mental health on RSFC in YPHIV remain understudied.

**Methods:**

We conducted a cross-sectional analysis of 155 participants (73 HIV-negative controls (HC), 82 (YPHIV) from the Cape Town Adolescent Antiretroviral Cohort Substance, Imaging and Mental Health (CTAAC-SIM) study. All underwent 3T anatomical and resting-state functional MRI scanning, mental health assessments, urine dipstick for commonly used substances and self-reported substance use screening using the Alcohol, Smoking and Substance Involvement Screening Test (ASSIST). Seed-based connectivity (SBC) analyses focused on the medial prefrontal cortex and 16 bilateral subcortical seeds (four striatal nuclei, amygdala, hippocampus, thalamus and ventral tegmental area). We conducted an analysis of covariance and multivariate linear regression to assess RSFC differences between HC with/without reported alcohol use and YPHIV with/without reported alcohol use. In addition, we explored the association between RSFC and anxiety and depression symptom scores within the different groups. We controlled for age, gender and education. All results were FDR-corrected for multiple comparisons.

**Results:**

In YPHIV with alcohol use compared to YPHIV with no alcohol use, we found significantly greater RSFC of the right caudate to temporal, occipital and cerebellar regions. In YPHIV (with/without alcohol use) and HC (with/without alcohol use), altered RSFC of the thalamus and amygdala were associated with higher depression symptoms but not anxiety.

**Conclusion:**

Alcohol use in YPHIV may be associated with alterations in subcortical RSFC as well as depression severity. Our findings are consistent with previously reported RSFC changes in emotion- and reward-related circuitry in HIV.

## Introduction

Youth living with perinatally acquired HIV (YPHIV) face unique neurodevelopmental challenges due to the lifelong effects of HIV infection and its treatment during critical neurodevelopmental phases (Laughton et al., 2013; Mellins and Malee, 2013). HIV infection alone has been associated with poor mental health outcomes in youth, with depression and anxiety being among the most common psychiatric disorders (Nosik et al., 2021; Vreeman et al., 2017). Additionally, participating in risky behaviours such as substance use increases in youth (Simon et al., 2022). The impact of substance use on brain development is particularly pronounced when use begins during adolescence (Weissman et al., 2015). Early substance use has been linked to altered functional connectivity between the regions of the reward system (i.e., nucleus accumbens (nAcc) and regions of control networks (i.e., prefrontal cortex (PFC)).

Most neuroimaging studies in YPHIV have investigated the association of HIV infection with structural rather than functional changes (Caceres et al., 2024; Hoare et al., 2020, 2018; Lewis-de Los Angeles et al., 2020, 2017). Consequently, evidence on the independent influence of HIV infection on functional connectivity mostly comes from studies of adults living with HIV. These studies showed an altered resting-state functional connectivity (RSFC) in subcortical regions, including striatal and limbic areas in adults with HIV compared to seronegative controls (Al-Khalil et al., 2024, 2023; Li et al., 2019; Ortega et al., 2015; Taebi et al., 2022; Wang et al., 2011). RSFC alterations of key regions that underpin reward processing (i.e., ventral tegmental area (VTA), nAcc, and medial PFC) have also been observed in adults with alcohol use (Administration (US) and General (US), 2016). On the other hand, disrupted RSFC of limbic areas such as the amygdala, hippocampus and thalamus have been observed in adults living with HIV and linked to increased depression symptom severity (Philippi et al., 2020). Taken together, evidence from adult studies indicates that HIV infection and substance use are associated with RSFC alterations in brain networks relevant to reward and emotional processing and regulation. However, the combined effects of HIV infection and alcohol use on RSFC in YPHIV remain largely unexplored.

Resting-state functional magnetic resonance imaging (rs-fMRI) provides a non-invasive method to probe intrinsic connectivity and is particularly suitable for studying neural circuits implicated in HIV, alcohol use, and mood symptoms (Jonathan D. Power et al., 2014). Yet the existing literature has largely focused on RSFC in adults with HIV or relied on region-to-region approaches that may overlook broader connectivity patterns (du Plessis et al., 2017; Hays Weeks et al., 2022; Lew et al., 2023; Thomas et al., 2013). Studying connectivity between selected regions of interest (ROI) may lead to overlooking how regions typically affected by HIV infection and substance use are functionally connected to other brain areas (Cole et al., 2010). Seed-based functional connectivity analysis selects a predefined ROI (the “seed”) and computes the correlation between the seed’s Blood-Oxygen-Level-Dependent (BOLD) time series and every other voxel in the brain, producing voxel-wise, whole-brain connectivity maps (Reid et al., 2017). Moreover, seed-based RSFC analysis has been shown to produce reliability comparable to traditional ROI-to-ROI approaches, while also providing the advantage of whole-brain mapping to capture broader network interactions. Although seed selection is hypothesis-driven, the voxel-wise nature of seed-based maps makes this approach particularly useful to explore functional circuits linked to HIV infection, alcohol use, and mental health. To our knowledge, seed-based RSFC analyses comparing YPHIV with and without alcohol use, and examining how these patterns relate to depression and anxiety symptoms, have not been conducted.

The Cape Town Adolescent Antiretroviral Cohort Substance, Imaging and Mental health (CTAAC-SIM) study provides an opportunity to examine the separate and combined associations of HIV and alcohol use with brain function. In this cross-sectional analysis, we address these gaps by investigating how HIV infection, alcohol use and mental health are associated with RSFC in youth. We aimed to examine group differences between YPHIV with/without alcohol use and HC with/without alcohol use, as well as the association of RSFC with symptoms of depression and anxiety within YPHIV (with/without alcohol use) and HC (with/without alcohol use). Our RSFC analyses focused on key subcortical regions: i.e., structures involved in reward anticipation (caudate, putamen, globus pallidum, VTA, nAcc, and mPFC), and limbic structures involved in emotional regulation and contextual processing (amygdala, hippocampus, thalamus).

## Methods

### Participants

Data from 189 participants were collected from the CTAAC-SIM, a follow-up study of the CTAAC cohort, which examines the effect of substance use and HIV infection on mental health of youth living with HIV who are on antiretroviral therapy (ART) from Cape Town, South Africa (Hoare et al., 2020, 2018). The inclusion criteria for CTAAC-SIM are: aged 15-24 years, having a confirmed diagnosis of HIV infection, being stable on ART for more than 6 months, and able to provide informed assent or consent. HIV-negative controls (HC) underwent an additional rapid HIV antibody test before enrolment to confirm negative status, and were matched for ethnicity, home language, years of education and annual household income. Participants were excluded if they had recent or ongoing medical conditions (e.g., infections, diabetes, epilepsy, or tuberculosis); any CNS-related diagnoses (e.g., meningitis, stroke, lymphoma); a history of head injury with loss of consciousness or skull fracture; perinatal complications (e.g., neonatal jaundice, birth-related hypoxia); or neurodevelopmental disorders unrelated to HIV. For this analysis, we included data from participants who underwent fMRI scanning and had information on alcohol use as well as assessment of depression and anxiety symptom severity. Figure S1 provides more details on the exclusion of participants at different stages of the analysis.

### Ethical approval

Participation in the CTAAC study was voluntary, and all participants signed a written informed consent form. The CTAAC SIM study was approved by the Human Research Ethics Committee (HREC) in the Faculty of Health Sciences at the University of Cape Town (UCT) with reference number 311/2021.

### Measures

#### Alcohol use

Alcohol use was self-reported using the Alcohol, Smoking and Substance Involvement Screening Test (ASSIST) (Humeniuk et al., 2010). ASSIST is a brief self-report, screening tool developed by the World Health Organization to assess substance use and related risks in primary health care settings (Assanangkornchai et al., 2015; Gryczynski et al., 2015; Newcombe et al., 2005). It assesses the use of a wide range of substances, including tobacco, alcohol, cannabis, cocaine, stimulants, sedatives, hallucinogens, inhalants, opioids, and others using an 8-item questionnaire(Humeniuk et al., 2010). ASSIST is a valid, comprehensive assessment tool that has been widely used and can reliably provide a risk assessment for substance use and guide appropriate interventions(Assanangkornchai et al., 2015; Gryczynski et al., 2015; Newcombe et al., 2005). Reported alcohol use in our sample was defined as a score of 1 and above and no use was defined as a score of 0 on the ASSIST. We used this approach as the majority of HC and YPHIV had lower ASSIST scores. A score above 10 on the ASSIST for alcohol use indicates medium or high risk for alcohol use requiring intervention. Similarly, reported cannabis use was defined as a score of 1 and above and no use was defined as a score of 0 on the ASSIST. Additional substance use was assessed using urine samples tested with the UberTest (PTY) Ltd, South Africa (DOA10DIP02:10 Panel Dip with EtG). The panel tests for 10 different drugs including alcohol (ethyl Glucuronide,EtG).

#### Depression and anxiety

For participants over 18 years of age (n = 144), we analysed scores of the Center for Epidemiologic Studies Depression Scale (CES-D) (Radloff, 1977) and the Beck Anxiety Inventory (BAI) (Beck et al., 2012). The CES-D scale has been validated across diverse demographic groups, including adolescents (Radloff, 1991). For participants under 18 years of age (n = 13), we analysed scores of the Beck Depression Inventory for Youth (BDI-Y) and the Beck Anxiety Inventory for Youth (BAI-Y) from the Beck Youth Inventories - Second Edition (BYI-II) (Beck and Beck, n.d.).

### Structural and rs-fMRI acquisition

Imaging of participants was done using a 3T Siemens Skyra scanner with a 32-channel head coil at the Cape Universities Body Imaging Center (CUBIC), University of Cape Town. For structural images, a T1-weighted multi-echo MPRAGE (MEMPRAGE) sequence was used with the following parameters: repetition time (TR) = 2530 ms, echo time (TE) = 1.69 ms, inversion time (TI) = 1100 ms, flip angle = 7°, voxel size = 1 mm isotropic, matrix = 224 × 224, and slice thickness = 1 mm. For the acquisition of rs-fMRI data, a multiband-accelerated gradient-echo EPI sequence was used with the following parameters: TR = 832 ms, TE = 34.6 ms, flip angle = 52°, voxel size = 2.4 × 2.4 × 2.4 mm^3^, multiband acceleration factor = 6, matrix = 90 × 90, 100% FOV.

### Rs-fMRI preprocessing and quality assurance

Digital Imaging and Communications in Medicine (DICOM) structural and functional files were converted to NIfTI format using dcm2niix tool from MRIcroGL (Li et al., 2016) to facilitate further analysis. Preprocessing of structural and rs-fMRI data was performed using the CONN Toolbox (Nieto-Castanon and Whitfield-Gabrieli, 2022; Whitfield-Gabrieli and Nieto-Castanon, 2012) and SPM (Friston et al., 2011). Functional and anatomical data were preprocessed using the default preprocessing pipeline (Nieto-Castanon, 2020a) in CONN which includes realignment with correction of susceptibility distortion interactions, slice timing correction, outlier detection, direct segmentation and MNI-space normalization, and smoothing. Functional and anatomical data were normalized into standard MNI space and segmented into grey matter, white matter, and cerebrospinal (CSF) tissue classes (Calhoun et al., 2017; Nieto-Castanon, 2022). Functional data were then smoothed using spatial convolution with a Gaussian kernel of 8 mm full width half maximum (FWHM). Outlier scans were identified using ART (Whitfield-Gabrieli et al., n.d.) as scans with framewise displacement (FD) above 0.5 mm or global BOLD signal changes (GSC) above 3 standard deviations per participant (Nieto-Castanon, 2022; Jonathan D. Power et al., 2014). We used FD of > 0.5 mm and > 25% of invalid scans per participant as exclusion criteria after preprocessing. To determine the percentage of invalid scans per subject, CONN uses both FD and GSC values for each participant to generate a second-level covariate of percentage of invalid scans (Morfini et al., 2023). Based on the proportion of invalid scans and FD values for each participant, those with > 25% invalid scans and FD > 0.5 were excluded from the analysis (n = 17). After completing the preprocessing, functional data were denoised using the standard denoising pipeline (Nieto-Castanon, 2020b) in CONN (Figure S2). The denoising pipeline performs two steps: 1) linear regression of potential confounders using aCompCor to remove noise from white matter and CFS areas, subject-motion parameters and identified outlier scans per participant (Behzadi et al., 2007; Friston et al., 1996; Jonathan D. Power et al., 2014), and 2) temporal band-pass filtering of frequencies below 0.008 Hz or above 0.09 Hz (Hallquist et al., 2013). Final results were thresholded using a combination of a cluster-forming p < 0.001 voxel-level threshold, and a familywise corrected p-FDR (False Discovery Rate) < 0.05 cluster-size threshold (Chumbley et al., 2010). Further details on the preprocessing steps are provided in the supplementary material.

### Seed-based connectivity analysis

We analysed subcortical regions of interest (ROI) associated with HIV, alcohol use, depression, and anxiety. Using the CONN Toolbox, we chose 17 seeds: mPFC, VTA, nAcc, putamen, caudate, globus pallidum, amygdala, hippocampus, and thalamus. Except for the mPFC, all seeds were analysed bilaterally. Seed-based connectivity (SBC) maps of the above-mentioned regions were estimated characterizing the patterns of functional connectivity with 27 HPC-ICA networks (Nieto-Castanon and Whitfield-Gabrieli, 2022) and Harvard-Oxford atlas ROIs (Desikan et al., 2006). The VTA seed masks were obtained from the probabilistic VTA atlas publicly available on Open Science Framework (Trutti et al., 2021). Functional connectivity strength was represented by Fisher-transformed bivariate correlation coefficients from a weighted general linear model (weighted-GLM (Nieto-Castanon, 2020a)), defined separately for each pair of seed and target areas, modelling the association between their BOLD signal timeseries. Further details on the seed-based connectivity analysis in CONN are provided in the supplementary material.

### Statistical analysis

We conducted all statistical analyses in the CONN Toolbox. Age, gender, education level (coded as grades corresponding to 1-1212 as in the South African schooling system), viral suppression (coded 0 = virally suppressed/< 200 copies/mL, 1 = non-virally suppressed > 200 copies/mL), and CD4 cell count were imported into CONN as second-level covariates. To perform between-group analysis, we created binary variables for four groups. Our first aim was to determine group differences in seed-based RSFC of ROIs. Based on previous research (Arienzo et al., 2020; Crane et al., 2018; Cservenka et al., 2014), we hypothesised that greater RSFC of key ROIs of the reward system (i.e., striatal nuclei and mPFC) and lower RSFC in limbic regions (i.e., hippocampus, amygdala and thalamus) to be observed in the presence of alcohol use in YPHIV. To assess this, we conducted an analysis of covariance (ANCOVA) in the CONN Toolbox. We controlled for the confounding effect of age, gender, education, viral suppression, and CD4 cell count for YPHIV. Our second aim was to investigate associations between seed-based RSFC of ROIs in YPHIV with and without alcohol use and severity of anxiety and depression symptoms. We ran the same analysis in HC. Previous findings on RSFC of limbic regions are heterogenous but evidence from systematic reviews suggests hypoconnectivity of limbic regions such as the thalamus, amygdala and hippocampus in anxiety and depression (Tse et al., 2023; Zugman et al., 2023). Therefore, we hypothesised higher anxiety and depression scores to be associated with lower RSFC of limbic regions in YPHIV and in HC, both with reported alcohol use. We conducted linear regression analysis in CONN using raw scores from CES-D and BAI as the outcome variable, RSFC of the selected ROIs as the explanatory variable, and controlling for age, gender, education, viral suppression, and CD4 cell count for YPHIV.

## Results

### Sample characteristics

The total final sample consisted of 155 participants, 73 of whom were HC and 82 were YPHIV. The mean age was 19.3 years for controls and 20 years for YPHIV. Based on ASSIST scores for alcohol use, HC had a mean score of 4.8 (range 0–26) and YPHIV of 4.9 (range 0–21). Table 1 details the demographic and clinical characteristics of the sample, scores of alcohol use, and scores of anxiety and depression. After excluding those with reported cannabis use, the sample included in the ANCOVA and regression modelling consisted of 110 participants, 60 HC and 50 YPHIV. We categorised four groups based on reported alcohol use only and excluded those who reported cannabis use to focus on the effect of alcohol on RSFC. The groups were: HC without reported alcohol or cannabis use (n = 33), HC with reported alcohol use only and no reported cannabis use (n = 27). YPHIV without reported alcohol or cannabis use (n = 34), YPHIV with reported alcohol use only and no reported cannabis use (n = 16). 13 HC and 32 YPHIV reported both alcohol and cannabis use (ASSIST score 1 and above for both) and were therefore excluded from the four defined groups. Patterns of drinking reported by youth were occasional social binge drinking or weekend drinking. Daily drinking was not reported.

### Associations of alcohol use with RSFC in HC

Comparison of RSFC of the 17 seeds between HC with and without reported alcohol use showed no significant differences between the two groups.

### Associations of HIV and alcohol with RSFC in YPHIV

**Figure 1:**
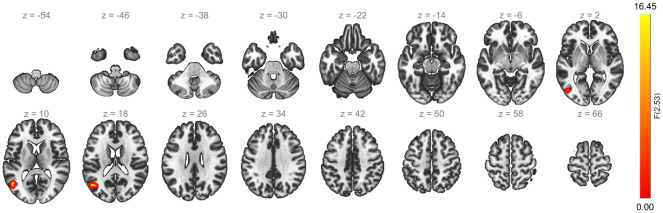
Significantly greater RSFC of the right caudate in YPHIV with reported alcohol use compared to YPHIV without reported alcohol use.

In YPHIV with alcohol use compared to those without alcohol use, we observed greater RSFC of right caudate to a cluster covering temporal and occipital regions as well as cerebellar areas. (Table S1, Figure 1).

### Association of RSFC with severity of anxiety and depression symptoms in YPHIV and HC with/without alcohol use

#### Anxiety

We did not observe any statistically significant association between RSFC and anxiety scores in the four groups.

### Depression

#### RSFC and depression scores in YPHIV with and without reported alcohol use

**Figure 2:**
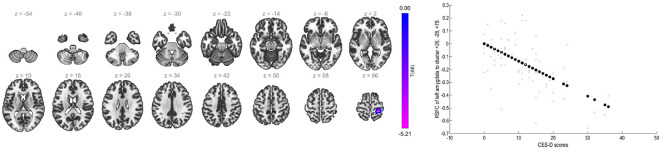
In YPHIV without reported alcohol use, lower RSFC of the left amygdala is associated with higher CES-D scores.

**Figure 3:**
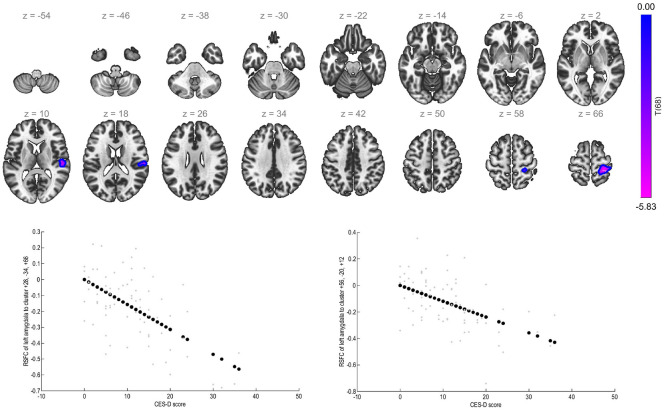
In YPHIV with reported alcohol use, lower RSFC of the left amygdala is associated with higher scores.

In both YPHIV with and without reported alcohol use, we found that lower RSFC of the left amygdala to clusters covering parietal, frontal, and temporal areas were significantly associated with higher CES-D scores (Table S2 and S3, Figure 2 and 3).

#### RSFC and depression severity in HC with and without reported alcohol use

**Figure 4:**
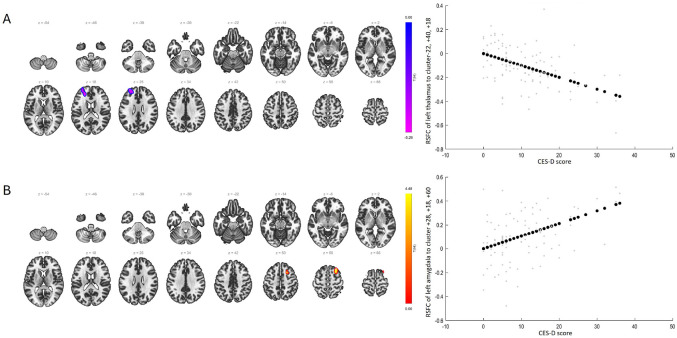
In HC without reported alcohol use: A) Lower RSFC of the left thalamus is associated with higher CES-D scores and B) greater RSFC of the left amygdala is associated with higher CES-D scores.

**Figure 5:**
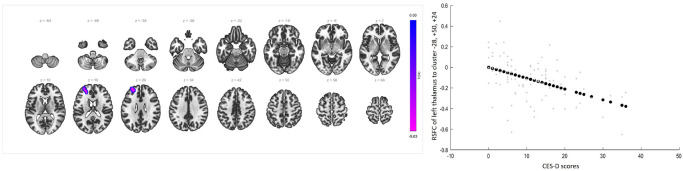
In HC with alcohol use, lower RSFC of the left thalamus is associated with higher CES-D scores.

**Figure 6:**
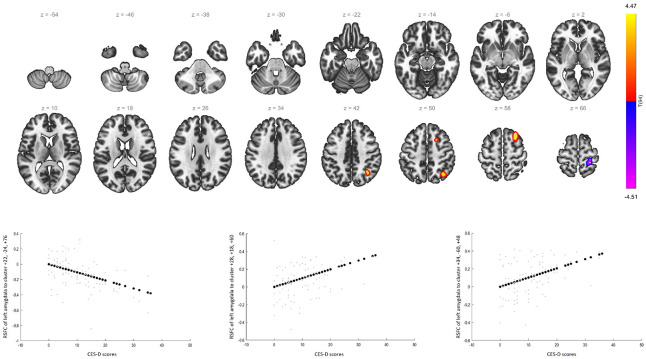
In HC with alcohol use, lower and greater RSFC of left amygdala is associated with higher CES-D scores.

In HC without reported alcohol use, we found that lower RSFC of the left thalamus and greater RSFC of the left amygdala to frontal regions were associated with higher CES-D scores (Table S4, Figure 4). In HC with reported alcohol use, we found a significant association between lower RSFC of the thalamus to frontal regions and higher CES-D scores (Table S5, Figure 5). Moreover, both lower and greater RSFC of the left amygdala to frontal, parietal, and occipital areas were associated with higher CES-D scores (Table S5, Figure 6).

## Discussion

This study explored the associations of alcohol use with altered RSFC of cortical and subcortical regions in youth living with perinatal HIV. In YPHIV with alcohol use compared to YPHIV with no alcohol use, we observed a greater RSFC of bilateral caudate to temporal, occipital and cerebellar regions. In YPHIV (with and without alcohol use) and HC (with and without alcohol use), altered RSFC of the thalamus and amygdala were associated with higher depression symptoms but not with anxiety symptoms.

The finding of a greater RSFC of the caudate to occipital areas, angular gyrus and middle temporal gyrus in YPHIV is consistent with previous rs-fMRI studies that showed greater caudate RSFC in both HIV infection and in alcohol use disorder (Nguchu et al., 2021; Plessis et al., 2014; Tolomeo and Yu, 2022). The caudate and striatal nuclei are part of the reward system and are involved in associative learning (Anderson et al., 2017) and inhibitory control (Nestler et al., 2010), which can be disrupted in substance use (Koob and Volkow, 2010). Our results suggest that additive effect of perinatal HIV in combination with alcohol use has a greater impact on striatal RSFC in youth. Interestingly, no significant RSFC differences were observed between HC with and without alcohol use, suggesting that occasional binge drinking may not alter RSFC in HC. In contrast, YPHIV may be more vulnerable to alcohol’s effects.

In YPHIV with and without reported alcohol use, lower RSFC of the amygdala to frontal, parietal, and temporal regions were associated with greater depression symptoms. Notably, HC without reported alcohol use showed greater RSFC of the amygdala to frontal areas, which was associated with higher depression scores. Greater fronto-amygdala RSFC is consistent with a previous finding from a meta-analysis of adults and adolescents with major depressive disorder (MDD) (665 patients and 546 controls) which demonstrated increased amygdala RSFC to frontal areas such as the ventromedial prefrontal cortex in MDD (Tang et al., 2018). The reverse findings in YPHIV in similar regions, suggest that HIV infection may play a role in modulating fronto-amygdala connectivity that is associated with depression in YPHIV.

We also found that in HC, regardless of alcohol use, higher depression scores were linked to decreased RSFC between the thalamus and frontal regions. This finding aligns with previous research indicating reduced thalamocortical connectivity in major depression (Zheng et al., 2023). Interestingly, thalamic RSFC did not seem to be associated with depression severity in YPHIV with or without alcohol use in this sample. Overall, our findings may suggest that subcortical neural pathways implicated in depressive symptoms, particularly of the amygdala and thalamus, are inversed between YPHIV and controls.

Our findings build on prior evidence from youth populations living with HIV. While previous rs-fMRI research on HIV and alcohol use in both adults and adolescents mainly focused on cognitive functions, our study provides neuroimaging evidence of functional alterations in YPHIV. Our results suggest that RSFC disruptions in key reward related regions can be detected in YPHIV with alcohol use prior to adulthood. This early emergence is particularly significant because adolescence is a period of rapid neural network refinement, during which circuits supporting emotion regulation, reward processing, and executive control are still maturing (Casey et al., 2008). Long-standing HIV infection, compounded by alcohol use, may disrupt this critical developmental trajectory, potentially leading to atypical connectivity patterns associated with lifelong disrupted self-regulation and decision making (Becker et al., 2011). Speculatively, in resource-limited settings, where HIV-related stigma and gaps in mental health care are prevalent (Nice et al., 2024), these neurodevelopmental disruptions may be further exacerbated, increasing vulnerability to persistent mental health and substance use challenges (Laughton et al., 2013).

There are several limitations. First, the sample size in the 4-group analysis (YPHIV with/without alcohol use and HC with/without alcohol use) is relatively small and may result in type II errors. However, our study provides preliminary evidence that merits further investigation. Second, alcohol use was assessed primarily by self-report, with objective verification for alcohol use on urine samples unavailable for all participants. Nonetheless, although the reported alcohol use score on the ASSIST being lower than 10 for most of our participants, and the pattern of alcohol use reported was social binge alcohol use over weekends or gatherings rather than daily drinking, our findings on altered RSFC may suggest that in youth with perinatal HIV, engaging in social alcohol use may be sufficient to affect RSFC, potentially forming maladaptive addiction-related pathways in the developing brain.

## Conclusion

In summary, we showed that RSFC of key brain regions implicated in reward processing and emotional regulation are significantly associated with alcohol use in YPHIV and that regardless of alcohol use, altered RSFC of limbic regions is related to greater depression symptoms. Looking forward, by increasing sample size and examining additional brain regions and resting-state networks may help track cortical and subcortical RSFC alterations in YPHIV with alcohol use, while integrating mental health assessment. Future work can further the understanding of the synergistic effects of HIV infection and alcohol use by conducting longitudinal analyses, integrating other mental health measurements as well as comprehensive immunological biomarkers to elucidate the full impact of these factors combined on the developing brain.

## Supplementary Material

This is a list of supplementary files associated with this preprint. Click to download.

• SupplementarymaterialrsYPHIV.docx

## Figures and Tables

**Table 1:** Details of demographic and clinical characteristics of the analysed sample, ASSIST scores for alcohol and cannabis use, as well as scores of anxiety and depression based on CES-D and BAI for controls and YPHIV.

	HC (n = 73)	YPHIV (n = 82)	T-test/Chi square p-value
**Age, mean ± SD**	19.3 ± 1.9	20.0 ± 1.8	0.02
**% Female gender (n)**	63% (46)	51% (42)	0.29
**% Education in grade 10-12 (n)**	67% (49)	63.4% (52)	0.07
**CD4% (mean)**	40.05%	29.02%	<0.0001
**% Viral suppressed (n)**	NA	56.1 % (46)	NA
**ASSIST alcohol score, mean ± SD**	4.8 ± 5.8	4.9 ± 6.4	0.95
**CES-D total score, mean ± SD**	12.4 ± 9	10 ± 6.8	0.07
**BAI total score, mean ± SD**	5.1 ± 7.4	3 ± 5.6	0.06

Abbreviations: ASSIST: Alcohol, Smoking and Substance Involvement Screening Test; CES-D: Center for Epidemiologic Studies Depression Scale; BAI: Beck Anxiety Inventory. Notes: Viral load: Based on the sample volume tested, the lower limit of detection is 20 RNA copies/ml. The linear range of this assay is 20 - 10,000,000 RNA copies/mL (1.3 - 7 log copies/mL).

## Data Availability

The datasets generated and/or analysed during the current study are not publicly available but can be shared by the corresponding author or study PIs upon reasonable request and approval of study PIs.
